# Using microsatellite data to estimate the persistence of field-level yield gaps and their drivers in smallholder systems

**DOI:** 10.1038/s41598-023-37818-2

**Published:** 2023-07-10

**Authors:** Ambica Paliwal, Balwinder-Singh, Shishpal Poonia, Meha Jain

**Affiliations:** 1 School for Environment and Sustainability, University of Michigan, Ann Arbor, MI 48109 USA; 2 International Livestock Research Institute, Nairobi, 00100 Kenya; 3 International Maize and Wheat Improvement Center (CIMMYT), New Delhi, 110012 India; 4 DPIRD, Government of Western Australia, 75 York Road, Northam, WA 6401 Australia

**Keywords:** Agroecology, Environmental impact

## Abstract

One way to meet growing food demand is to increase yields in regions that have large yield gaps, including smallholder systems. To do this, it is important to quantify yield gaps, their persistence, and their drivers at large spatio-temporal scales. Here we use microsatellite data to map field-level yields from 2014 to 2018 in Bihar, India and use these data to assess the magnitude, persistence, and drivers of yield gaps at the landscape scale. We find that overall yield gaps are large (33% of mean yields), but only 17% of yields are persistent across time. We find that sowing date, plot area, and weather are the factors that most explain variation in yield gaps across our study region, with earlier sowing associated with significantly higher yield values. Simulations suggest that if all farmers were able to adopt ideal management strategies, including earlier sowing and more irrigation use, yield gaps could be closed by up to 42%. These results highlight the ability of micro-satellite data to understand yield gaps and their drivers, and can be used to help identify ways to increase production in smallholder systems across the globe.

## Introduction

Food production may have to increase by up to 70% by mid-century to achieve global food security^1^. One way to increase yields is to close existing yield gaps, which are defined as the difference between current yields and the potential yields that could be achieved under ideal management^2,3^. Closing yield gaps is particularly important in smallholder systems, where yield gaps are large^4^ and food demand is likely to increase the most over the coming decades^1^. Correctly estimating yield gaps and their drivers is challenging in smallholder systems due to a lack of agricultural production data, particularly at the field scale^5^. Conventional methods of collecting field-level yield estimates, such as crop cuts, are time and cost intensive^6^. Satellite data have been shown to be a valuable tool for estimating yields, and quantifying yield gaps and their drivers at the landscape scale^7–9^. Yet, historically available imagery, such as Landsat (30 m) and MODIS (250 m), which have primarily been used to map yield gaps in smallholder systems^10,11^ are likely too coarse in spatial resolution to map individual field-level yields^12^.

Over the last five years, new micro-satellite data have become readily available and have shown promise for mapping yields at the field scale^5,12^. Despite the availability of such data since 2015, to date microsatellite data have not been used to map yield gaps and their drivers over multiple years. Yet, understanding the persistence of yields and yield gaps through time provides insights into the possible drivers of yield gaps^9^. For example, if yields are persistent, this suggests that there may be some consistent infrastructural (e.g., irrigation access) or biophysical (e.g., soil type) factors that largely explain yield gaps in these systems. On the other hand, if yields are not persistent, meaning that different fields have the largest yield gaps in different years, this suggests that there may be some time varying factor, such as weather (e.g., inter-annual rainfall variability) or management (e.g., sowing date), that primarily explain yield gaps^13^. Such analyses can provide critical information about what potential interventions may effectively close yield gaps at the field scale in these systems^14^.

Here we show that microsatellite data are able to accurately map field-level yields, and quantify yield gaps and persistence, at regional scales and over multiple years in smallholder systems. We also show that these data can also be combined with ancillary data on management, weather, and biophysical factors to identify which factors are the most important in explaining field-level yield gaps over multiple years. We focus our study in wheat systems in eastern India, where yield gaps are large and field sizes are especially small^10,15^ (< 0.3 ha). We specifically use SkySat and PlanetScope data (~ 3 m resolution) to map field-level yields from 2014 to 2019 and assess the magnitude of yield gaps and their possible drivers. Using this information, we then assess how much yield gaps can be closed under ideal management conditions, providing invaluable insights for how much yields may be able to increase over the coming years. While our study is focused on wheat systems in eastern India, our approach can be used to quantify yield gaps, yield persistence, and their drivers in smallholder systems across the globe.

## Methods

### Study area

**Figure 1:**
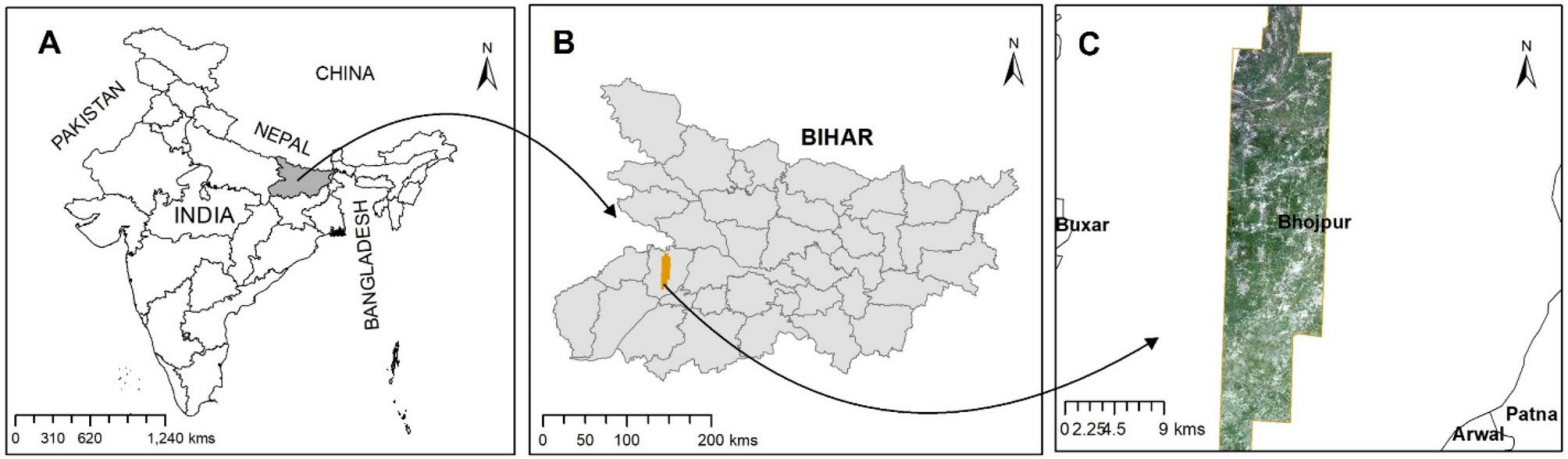
Map of India with Bihar highlighted in gray (Panel A) and the extent of the study region in Arrah district, Bihar highlighted in orange (Panel B). Panel C shows the PlanetScope image from February 15, 2018. This map is generated using ArcGIS Desktop 10.8 Version 10.8.0.12790 (http://www.esri.com).

We conducted our study in Arrah district, Bihar, India (25.47^◦^N, 84.52^◦^E), which is in the eastern portion of India’s main grain belt, the Indo-Gangetic Plains (IGP) (Fig. 1). Our analyses are focused on an 8 × 16 km^2^ area where we had access to SkySat and PlanetScope imagery over multiple years. The region is dominated by smallholder farms (< 0.3 ha)^12^ and over 80% of the land area is under agriculture. Most farmers in the region follow a rice–wheat cropping system, with rice planted during the monsoon (kharif) growing season and wheat planted in the winter (rabi) growing season. Our analyses focus on wheat as previous studies have shown that yield gaps for wheat are large in this region and are expected to increase given the negative impacts of warming temperatures on wheat yield^4,10^. The wheat growing season spans from early November to mid-April, and wheat management varies widely across farms resulting in significant across-farm heterogeneity in yield^12^. For example, wheat sowing dates vary from early November to early January and farmers also vary the number of irrigations applied throughout the growing season (ranging from 1 to 3 irrigations^12^).

### Field data collection and processing

We collected crop cut data from 271 randomly-selected wheat fields from 2014–15 to 2018–19, with the number of fields ranging from 36 to 79 in a given year (details provided in Table S1). Crop cuts are considered to be the gold standard for yield estimation, and are widely used to estimate crop yield on the ground^16^. It is important to note that different fields were visited in different years, meaning that repeat samples were not collected for the same field through time. To collect crop cut data, the field team visited each of the 271 fields at the time of crop harvest (in the month of April) and randomly selected subplots to harvest from each of the farmer’s fields (details in Table S1), as randomly selected subplots have been shown to approximate full-field crop cuts well^17^. The team harvested the crop from these subplots, threshed the sample, dried the sample, and weighed the grain on site. We then averaged the yields from each subplot for each field to estimate the average yield per hectare for the full field. In addition, the field team collected five GPS points, one from the field’s center and four from the corners of each field. For years 2014–15 and 2015–16, we also conducted a survey about farmers’ management practices in the field for that year in December (close to the timing of planting) and in April (after crop harvest). The survey included questions about management factors shown to be important for explaining wheat yields in the previous literature, including sow date and the number of irrigations throughout the season. This work was considered exempt for Human Subjects Research by the University of Michigan Institutional Review Board (HUM00156479, HUM00128955, HUM00120778). All the methods reported below were performed according to the relevant guidelines and regulations.

To derive field polygon boundaries from the five GPS coordinates for each field, we used the rgeos^18^ and sp^19^ packages in R Project Software Version 4.1.0^20^. We overlaid all field polygons over high-resolution imagery from Google Earth (https://www.google.com/earth/), and adjusted field polygons to match field boundaries that were visible in the high-resolution imagery^12,21^. We then linked all associated yield and management factors with each polygon, and used the resulting shapefile for all subsequent analyses.

### Satellite yield estimation

We used SkySat (2 m) imagery for the 2014–2015 and 2015–2016 growing seasons, and PlanetScope (3 m) imagery for the 2016–2017 to 2018–2019 growing seasons to estimate wheat yield. The number of images and specific dates used varied across years depending on image availability and cloud cover (details provided in Table S2). We assessed image availability by searching the PlanetScope API (www.planet.com) for all available images for our study site (Fig. 1) from November 1 to April 15 for each year’s growing season. We then visually inspected all available images and selected only those images that were cloud free. Since multiple tiles encompassed our study area, we mosaicked tiles into one image that covered the full study area extent using histogram-matching of overlapping areas in ENVI Software. SkySat imagery were provided as top of the atmosphere reflectance, so we corrected imagery to surface reflectance by stretching histograms to match distributions of each band as seen in cloud-free, surface reflectance corrected Landsat 7 and 8 imagery obtained from Google Earth Engine^22^ (GEE). Specifically, images were matched to cloud-free Landsat scenes from the closest available image date, and if a cloud-free Landsat scene was not available within two days of a given SkySat scene, we used a date-weighted average of the histograms from the two closest Landsat scenes before and after each available SkySat date (more details provided in Jain et al.^12^). All PlanetScope imagery were provided as surface reflectance corrected data, and thus all images were used directly without additional corrections.

SkySat and PlanetScope have blue (450–515 nm, 450–515 nm), green (515–595 nm, 500–590 nm), red (605–695 nm, 590–670 nm), and near infrared (NIR, 740–900 nm, 780–860 nm) bands. Using these bands, we calculated the green chlorophyll vegetation index for each image (GCVI) (Eq. 1) as previous studies have shown that GCVI has a linear relationship with the leaf area index for wheat^23^.

1 $$ {\text{GCVI }} = \, \left( {{\text{NIR}}/{\text{green}}} \right) \, - { 1} $$

**Figure 2:**
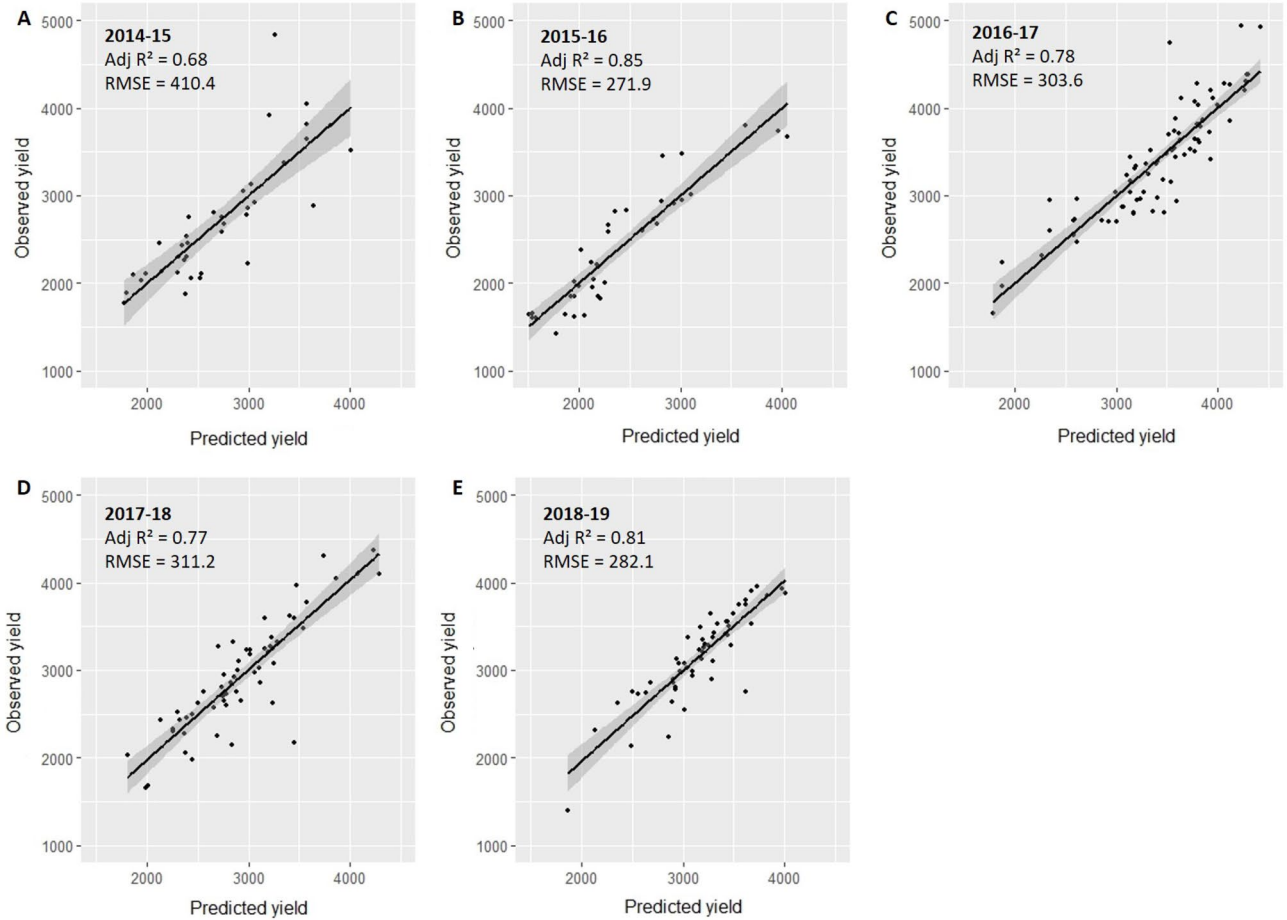
Plot level prediction accuracy for each year 2014–2015 to 2018–2019 (Panels A through E).

We then extracted the mean GCVI for each field polygon for each image date in all years, and these mean GCVI values were used to create our yield estimation model. We predicted yield using random forest regressions, where each year’s crop cut data were used to train individual random forest models for each year (Eq. 2)

2 $$ {\text{Crop cut yield}} \sim {\text{GCVI}}_{{1}} + {\text{ GCVI}}_{{2}} \ldots \, + {\text{ GCVI}}_{{\text{n}}} $$

where crop cut yield (kg/ha) is the observed yield estimated using crop cuts for each polygon, and GCVI_1_ to GCVI_n_ are the mean GCVI values for each polygon for each image date (n) within a given growing season. For each year, the estimated random forest model was used on the stacked GCVI raster layer for the respective year to predict yield values. Through previous work, we have found that the models that lead to the highest yield prediction accuracies are the ones that use GCVI data throughout the growing season^10,12^. In particular, it is helpful to have images from the early growing season and near the timing of peak greenness^10^. We used a similar approach in this study, where we used GCVI from all available image dates (stack GCVIs) to predict yield and get similar accuracies to those that we have found in our other yield mapping papers. Though GCVI values from different dates were correlated in some models (Table S3), this does not impact our results. This is because random forest regressions are robust to multicollinearity, particularly for prediction, and we were not interested in identifying the relative importance of each GCVI value, which is more likely to be impacted by multicollinearity^24^. Furthermore, though random forest is robust to overfitting, we ensured that our models did not overfit the data by running a fivefold cross validation analysis where we used 70% of the data for training and 30% of the data for testing. We find that prediction accuracies are similar between training and test datasets (Table S4), suggesting that our models are not overfitting the data. However, the random forest yield prediction values did not fall consistently on the one-to-one line when plotting predicted versus observed yield values (Figure S1). Furthermore, percent bias (PBIAS) was not equal to zero (Figure S1), suggesting that there is systematic over or underestimation of yields in each year. Thus, to correct for this systematic bias, we conducted a second step, where we regressed the observed crop cut yields on the random forest estimated yields using a linear regression (Eq. 3).

3 $$ {\text{Crop cut yield}} \sim \beta_{0} + \, \beta_{{1}} {\text{RF estimated yield }} + \, \varepsilon $$

where crop cut yield (kg/ha) is the observed yield estimated using crop cuts for each polygon, RF estimated yield (kg/ha) represents the mean predicted yield for each polygon derived from the random forest model from the first step (Eq. 2), and ε represents the residual error. To calculate mean satellite yield, we took the mean value of all pixels within each field’s polygon. We then applied the coefficients from Eq. 3 to the full raster stack to correct predicted random forest yields at the pixel scale across the study site. It is important to note that this correction (Eq. 3) was conducted separately for each of the five years. We validated our satellite yield estimates at the polygon scale by comparing estimated yields after the two-step approach with observed crop cut yields at the field scale (Fig. 2). Accuracy was evaluated using R^2^ and root mean squared error (RMSE) on the full dataset also used for training, which is a common approach in satellite yield estimation when there are a small number of crop cut samples available^5,12^.

### Yield gap estimation and persistence

To estimate yield gaps (YG), we subtracted the mean yield (Ymean ) for each polygon from yield potential (Yp) for each year (y; Eq. 4).

4 $$ {\text{YG}}_{{\text{y}}} = {\text{ Yp}}_{{\text{y}}} {-}{\text{ Ymean}}_{{\text{y}}} $$

where YG_y_ equals the yield gap (kg/ha) for each polygon in year y, Yp_y_ represents the yield potential (kg/ha) for all polygons in a given year y, and Ymean_y_ represents the mean yield (kg/ha) for each polygon in a given year y. We defined Yp_y_ as the 95th percentile yield value found in each year’s satellite estimated yield raster for the study region after masking out non-cropland pixels using land cover classes from the Global Land Cover product^25^. Previous studies have suggested that such empirically estimated Yp better captures realistic economically-achievable yields that consider real-world infrastructural, management, and economic constraints, which are not well accounted for in modelled estimates of Yp^9^. We calculated Ymean using the mean satellite estimated yield for each polygon for each year, as crop cut yield values were not available for each field in each year.

We measured yield persistence in two ways. First, we estimated how consistently fields were relatively high or low yielding by conducting a decile analysis developed in Lobell^9^. Specifically, all 271 fields were categorized into one of ten deciles based on their yield rank using 2014–15 satellite estimated yields. Keeping each field within its original categorized decile from 2014–15, we plotted boxplots of all yields seen for all fields across all remaining years (2015–16 to 2018–19). If yields are persistent, we would expect there to be little overlap between boxplots across decile values, as the lowest yielding fields would always be low yielding and the highest yielding fields would always be high yielding. However, if there is a large amount of boxplot overlap across deciles, this suggests that there is variability in yield through time. Second, we quantified the percent of yield variation that was persistent through time using methods from Lobell et al.^26^. Specifically, for the highest yielding decile of fields found in 2014–15, we calculated the fields’ average anomaly from the study site mean yield in 2014–15. For these same fields, we then calculated the fields’ average anomaly compared to the study site mean for all subsequent years (2015–16 to 2018–19). By comparing yield anomalies from 2014–15 with yield anomalies for all remaining years, we gain an understanding of the amount of yield persistence from 2014–15 across later time periods.

### Drivers of yield gaps and the ability to close yield gaps

To understand which factors most influence yield gaps, we conducted random forest regressions where we regressed yield gap estimates for each year on a suite of management, weather, and biophysical variables that have been suggested to be important drivers of yield gaps in the previous literature (Eq. 5). Specifically, for management variables we considered wheat sowing date and the number of irrigations applied, for weather variables we considered average temperature and total rainfall within each winter season, and for biophysical variables we considered soil nitrogen and soil organic carbon.

5 $$ YG_{y} \sim \beta_{0} + \beta_{1} {\text{DOS}}_{y} + \beta_{2} {\text{Irrigation}}_{y} + \beta_{3} {\text{AvgTemp}}_{y} + \beta_{4} {\text{Tot}}\_{\text{Rain}}_{y} + \beta_{5} {\text{Nitrogen }} + \beta_{6} {\text{Soil}}\_{\text{Org}}\_C \, + \beta_{7} {\text{Plotarea }} + \, \varepsilon $$

where YG_y_ represents the yield gap (kg/ha) calculated for each year for each field (from Eq. 4), DOS_y_ represents the sowing date of wheat (days since November 1) for each field in each year, Irrigation_y_ represents the number of irrigations (ranging from 1–3) applied to each field during the wheat growing season in each year, AvgTemp_y_ represents the average temperature (°C) for each polygon in each year, Tot_Rain_y_ represents the total amount of rainfall (mm) for each polygon in each year, Nitrogen represents mean soil nitrogen (cg/kg) for each field across all years, Soil_Org_C (dg/kg) represents mean soil organic carbon (SOC) for each field across all years, Plotarea represents area of the field, and ε represents error. We calculated variable importance for our random forest regression (Eq. 5) by examining the mean decrease in accuracy (%IncMSE) over all out-of-bag cross validated predictions when each variable was permuted.

We obtained sowing date and irrigation information from management surveys that were conducted in 2014–15 and 2015–16. Given that we only had management variables available for these two years, we restricted our analyses (Eq. 5) to only these two years. We calculated average temperature using temperature data from Terra Climate^27^; specifically we calculated mean temperature for each month (November to April) for each year (2014–15 to 2018–19) using the mean of monthly maximum and minimum temperature. We calculated total rainfall as the sum of monthly rainfall from November to April using monthly precipitation data from Terra Climate^27^. Finally, we calculated soil nitrogen and SOC using World Soil Information Service (WoSIS) global raster data^28^. Weather and soil raster data were extracted as the mean value for each polygon using the raster package^29^ in R Project Software 4.1.0^20^. More details about each dataset, including their source and resolution are included in TableS6.

Finally, we ran simulations to quantify how much yield gaps could be closed if all farmers adopted optimal management strategies. For this analysis, we focused on the two management variables considered in our analyses (Eq. 5), sowing date and number of irrigations applied. To identify what management values were optimal, we examined the partial dependence plots of sow date and irrigation and identified which values were associated with the smallest yield gaps. Partial dependence plots show the marginal effect of each feature on the predicted outcome from our random forest analysis (Eq. 5). Based on the partial dependence plots, we found that a sowing date of November 12 and three irrigations were associated with the lowest yield gaps. In our scenario analysis, we therefore altered all sowing dates to be November 12 and all irrigations to equal 3, and we predicted what yields would be for each field using our random forest model (Eq. 5). To estimate how much yield gain could be achieved, we quantified the difference between this predicted yield value under optimal management and Y mean_y_. All analyses were done using the Random Forest^30^ and partial dependence plot^31^ packages in R Project Software 4.1.0^20^.

## Results

### Accuracy of satellite estimated yields at the field scale

Overall, we find that micro-satellite data can accurately map yield at the field scale across multiple years. While accuracies varied from year to year, all years resulted in moderate to high validation accuracies (R^2^ values range from 0.68 to 0.85), suggesting good fit with observed yield values (Fig. 2A-E). RMSE values were also moderate, ranging from 272 kg/ha to 410 kg/ha across the five years.

### Magnitude and persistence of yield gaps

**Figure 3:**
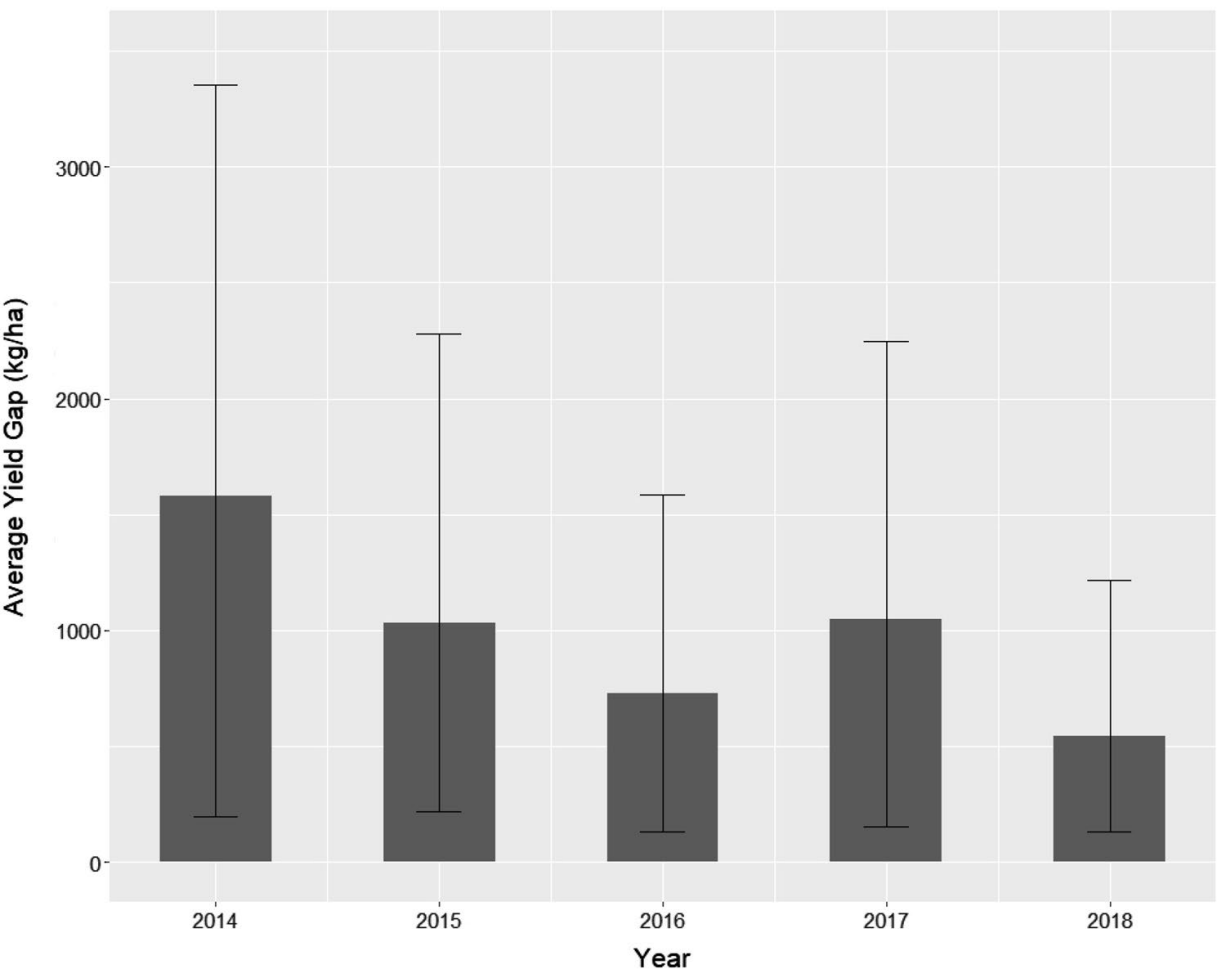
Average yield gap with 95% confidence intervals for each year.

**Figure 4:**
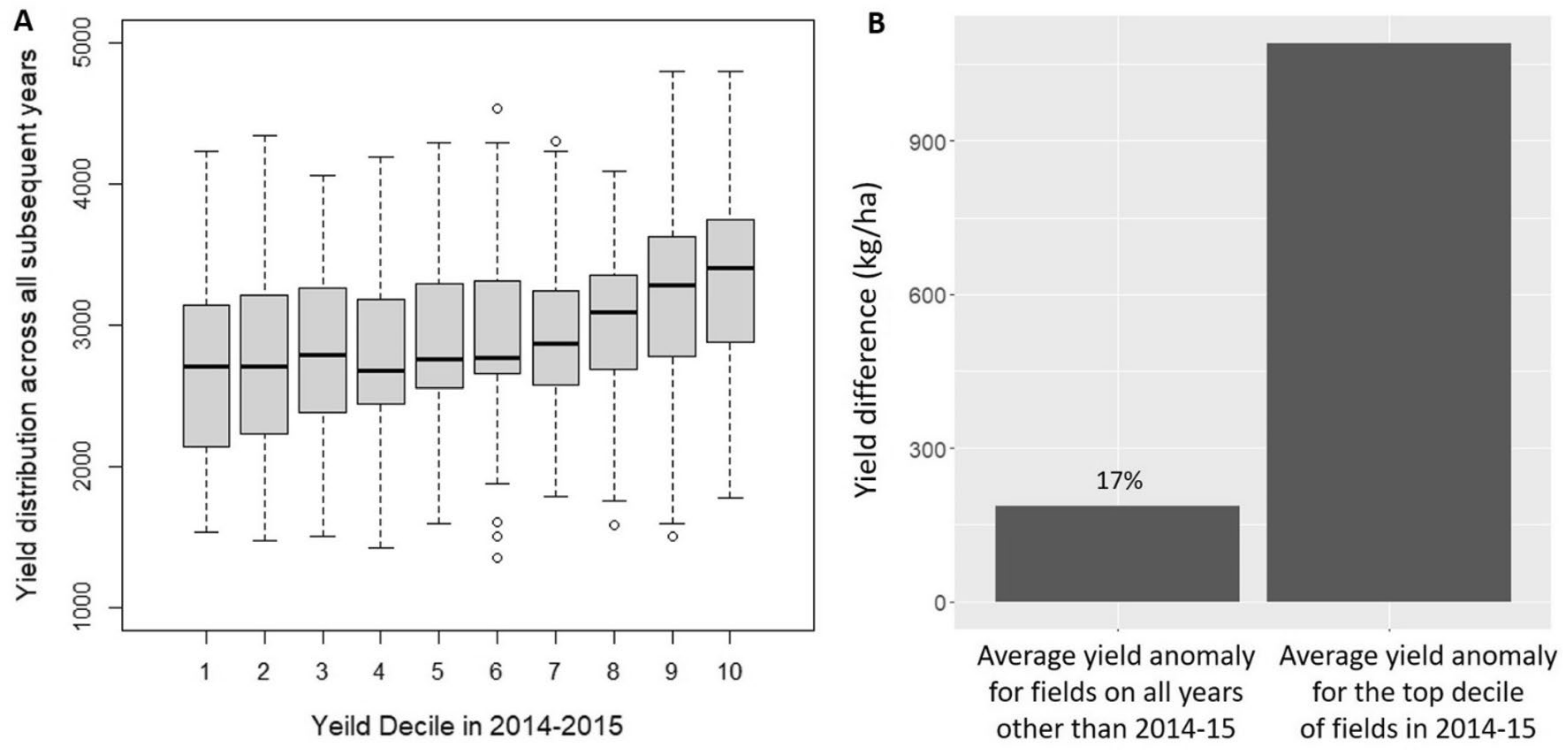
Yield distribution for the ten decile groups defined using 2014–15 yield estimates (Panel A). Average yield anomaly for the top decile of the fields in 2014–15 and average yield anomaly for the fields for all remaining years (2015–16 to 2018–19, Panel B).

We find that overall yield gaps are large, with an average value of 985 kg/ha across all polygons and all years. This is equal to 33% of mean yield values. The average yield gap varies from year to year, ranging from 543 to 1579 kg/ha (Fig. 3). Considering persistence, we find that yields are somewhat persistent through time and the level of persistence likely varies for low versus high yielding fields. Specifically, we find that there is high overlap in our decile boxplot analysis, particularly for lower decile values (Fig. 4A). This suggests that fields that are the lowest yielding in 2014–15 are not consistently low yielding across the timeframe of our study. There is, however, a positive increase in yields across decile values, and less overlap in boxplots for higher decile bins, suggesting that there is some yield persistence across time, particularly for higher yielding fields. Considering the amount of persistence that occurs for the highest yielding fields, we find that 17% of yield anomalies persist from 2014–15 to later time periods (Fig. 4B). To provide an overview of the yields per year, we have provided the descriptive statistics of yields per year at the field level in Table S5.

### Drivers of yield gaps and the ability to close yield gaps with ideal management

**Figure 5:**
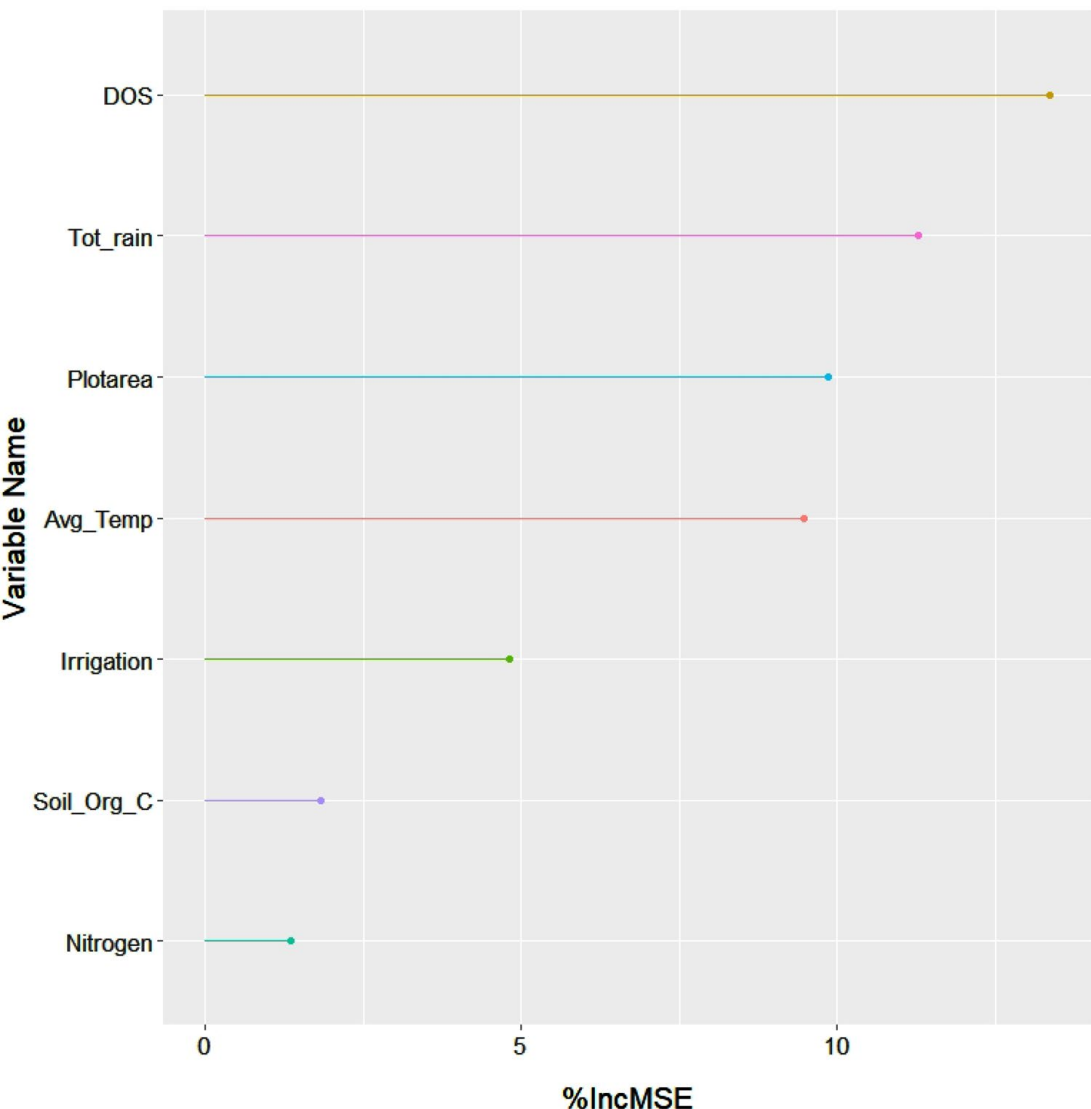
Variable importance plots for the management, weather, and biophysical factors considered to explain yield gaps for the years 2014–15 and 2015–16.

**Figure 6:**
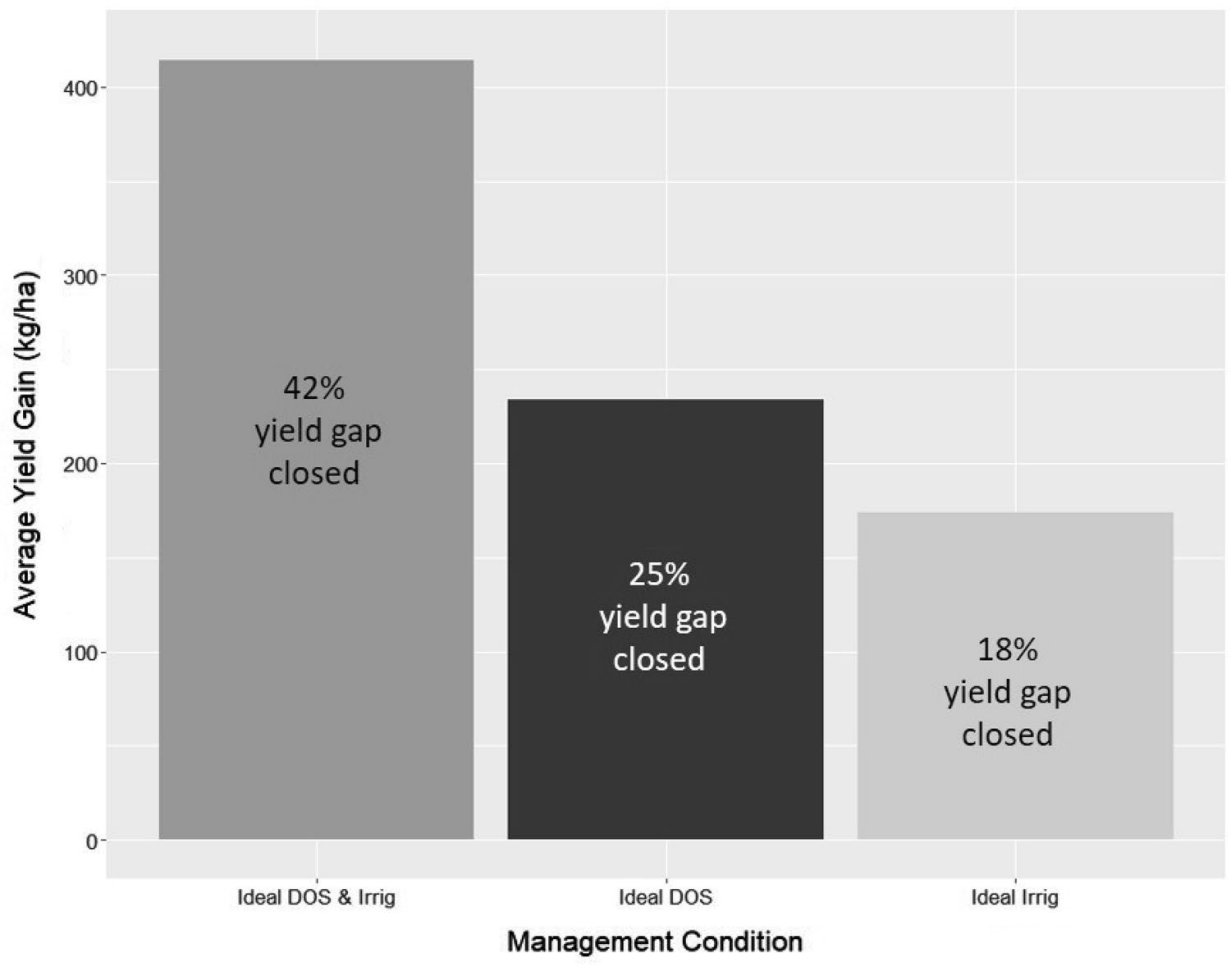
Average yield gain under three ideal management conditions (Ideal DOS represents sowing on November 12, and ideal irrig represents three irrigations).

When considering which biophysical, weather, and management factors drive yield gaps, we find that weather and management variables are the most important factors explaining variation in yield gaps (Fig. 5). Our model shows that amongst all variables considered, sowing date is the variable that explains the most variation in yield gap. Weather variables and plot area are also found to be important explanatory factors of yield gap, with average temperature and total rainfall explaining similar amounts of variation (Fig. 5). These results are robust to the use of disaggregated climate data, specifically monthly GDD and monthly precipitation values (Figure S2). Considering partial dependence plots of the management variables considered in our study, we find that later sowing dates are associated with larger yield gaps (Figure S3A) and November 12th is the sowing date associated with the lowest yield gap. Furthermore, yield gaps decrease as more irrigations are applied, with the lowest yield gap seen with three irrigations (Figure S3B). Inputting these ideal management factors (November 12, three irrigations) into our scenario analysis, we find that yields could be increased on average by 414 kg/ha across all fields, which is 42% of the estimated yield gap in this region (Fig. 6). Shifting only the sowing date to November 12 could close the yield gap by 25% and only optimizing irrigation could close the yield gap by 18% (Fig. 6).

## Discussion and conclusion

We find that microsatellite data can accurately map field-level yields in smallholder systems, and these data can be used to understand the magnitude of yield gaps, yield persistence, and the drivers of yield gaps at the landscape scale. This is exciting given that previous remote sensing studies that have focused on yield gap and persistence analyses in smallholder systems have relied on coarser resolution imagery (e.g., Landsat) that are unable to resolve yields at the field scale^12^.

Using micro-satellite data, we find that yield gaps are large on average (985 kg/ha), though the magnitude of yield gaps vary from year to year (Fig. 3). Our yield gap estimates are mostly smaller than those found in previous studies for the region, likely because we used an empirically derived estimate of economic yield potential (Yp). Specifically, by using the 95% percentile observed yield as our estimate of Yp, we are identifying the highest yielding field that exists given currently available inputs, soil health, and management practices. Previous studies have largely used crop model simulations or yields obtained from on-farm trials to estimate Yp^32–34^. When using crop model simulations or on-farm trials, ideal inputs and management practices are used (e.g., ideal sowing date, ideal input use), leading to a much larger estimate of Yp. For our study region, previous studies that have used modelled estimates of Yp find yield gaps that are about double those found in our study, ranging from 1500 to 2000 kg/ha. Yet such estimates of Yp do not account for existing economic and/or infrastructural constraints in the system that are difficult to alleviate, and thus represent long-term, idealized potentials. It is important to note that by calculating yield gaps for each year, we are examining the magnitude and causes of yield gaps while controlling for inter-annual variability in weather.

Our yield gap estimates are similar to those found in a previous study^10^ that used Landsat satellite data to map wheat yield gaps across the Indo-Gangetic Plains (IGP). This is likely because this study used a similar approach to quantify Yp and associated yield gaps. Considering yield persistence, we find that yields are 17% persistent in this region, and yields seem to be more persistent for fields with higher-than-average yields. This suggests that systemic factors that consistently vary across farms, such as differences in soil quality and farmer skill, will also need to be addressed to close yield gaps in this region, particularly for the highest yielding fields.

When analyzing the drivers of yield gaps, we find that sowing date, plot area, and weather are the factors that most explain variation in yield gap in our study region. Plot area is likely capturing the effect of improved management in larger fields that are typically owned by wealthier farmers^35,36^. The importance of sowing date has been highlighted by previous studies^10,37,38^, with farmers who sow earlier experiencing higher yields. This is because wheat is one crop that is particularly negatively impacted by heat stress that occurs at the end of the growing season during the time of grain filling^39^. If farmers are able to sow their wheat earlier, allowing the crop to mature prior to heat stress at the end of the growing season, negative yield impacts can be reduced^40,41^. We find that if farmers are able to sow their wheat earlier, they will be able to close yield gaps by 25% (Fig. 6). Furthermore, if farmers are additionally able to increase the amount of irrigation they use to three irrigations, yield gaps can be closed by 42%.

While this is a substantial amount of the yield gap, it must be noted that the adoption of these ideal management strategies may not be possible for all farmers. Previous studies have suggested that while farmers are aware of yield gains associated with earlier wheat planting, they are often constrained on when they can plant based on irrigation availability, monsoon rainfall patterns, and decisions made in the prior monsoon growing season^38,42^. Several policies may help advance sowing and increase irrigation use across farmers. The first is providing farmers access to low-cost groundwater irrigation, possibly through the promotion of solar pump technologies ^43^. There is scope to sustainable increase groundwater use in this region, but farmer use of groundwater is limited due to the reliance on costly diesel pumps ^38^. Second, the use of zero tillage has been shown to advance sow date by up to two weeks in the region, and policies that enhance the uptake of zero tillage, such as increased subsidies and the promotion of service providers, could help farmers advance sowing date^38,44,45^.

While our study is, to our knowledge, the first to map yield gaps and persistence in this landscape using microsatellite data, there are several important limitations. First, crop cuts were collected using different data collection protocols in each year (Table S1) and the same fields were not re-measured year after year. This is because we used data that were collected for several different research projects that were managed by different field teams and had different goals. We tested, however, if the method of crop cut used influenced our satellite yield estimates and find that there is no significant effect (Table S7). Second, our study examined a limited number of management variables that may influence yield gaps, namely sowing date and irrigation, and future work would benefit from including additional management strategies that have been shown to be important in the literature, such as sowing method and fertilizer use^21,44^.

In conclusion, our study highlights the ability of microsatellite data to map yield gaps, yield persistence, and the drivers of yield gaps in smallholder farming systems at the field scale. We show that our yield gap estimates are for the most part smaller than those reported in previous studies, as we are able to better capture economic yield potential using empirically driven estimates. We find that yields are somewhat persistent in the region, and that yield gaps can be closed by up to 42% if farmers are able to adopt ideal management strategies. To our knowledge this is the first study where microsatellite data have been used to assess yield gaps over multiple years in smallholder systems. While our study is focused in one region of India, we believe that microsatellite data can be similarly beneficial for yield gap analysis in other smallholder systems across the globe.

## Supplementary Information

Supplementary Information.

## Data Availability

The data that support the findings of this study are available from the corresponding author upon reasonable request.
