# Supplementary material for: Using microsatellite data to estimate the persistence of field-level yield gaps and their drivers in smallholder systems
Source: Sci Rep. 2023 Jul 10;13:11170. doi: 10.1038/s41598-023-37818-2 (PMC10333286; doi:10.1038/s41598-023-37818-2)
Supplement: Supplementary file 1 — Supplementary Information. [file 41598_2023_37818_MOESM1_ESM.doc]

**Supplementary Information**

**Using Microsatellite Data to Estimate the Persistence of Field-level Yield Gaps and Their Drivers in Smallholder Systems**

Ambica Paliwal^1,2*^, Balwinder-Singh^3,4^, Shishpal Poonia^3^, Meha Jain^1^

^1^ School for Environment and Sustainability, University of Michigan, Ann Arbor, MI 48109, United States of America

^2^ International Livestock Research Institute, Nairobi 00100, Kenya

^3^ International Maize and Wheat Improvement Center (CIMMYT), New Delhi110012, India

^4^ DPIRD, Government of Western Australia, 75 York Road, Northam, WA 6401, Australia

*Corresponding author: Ambica Paliwal [ambica@umich.edu](mailto:ambica@umich.edu)

**
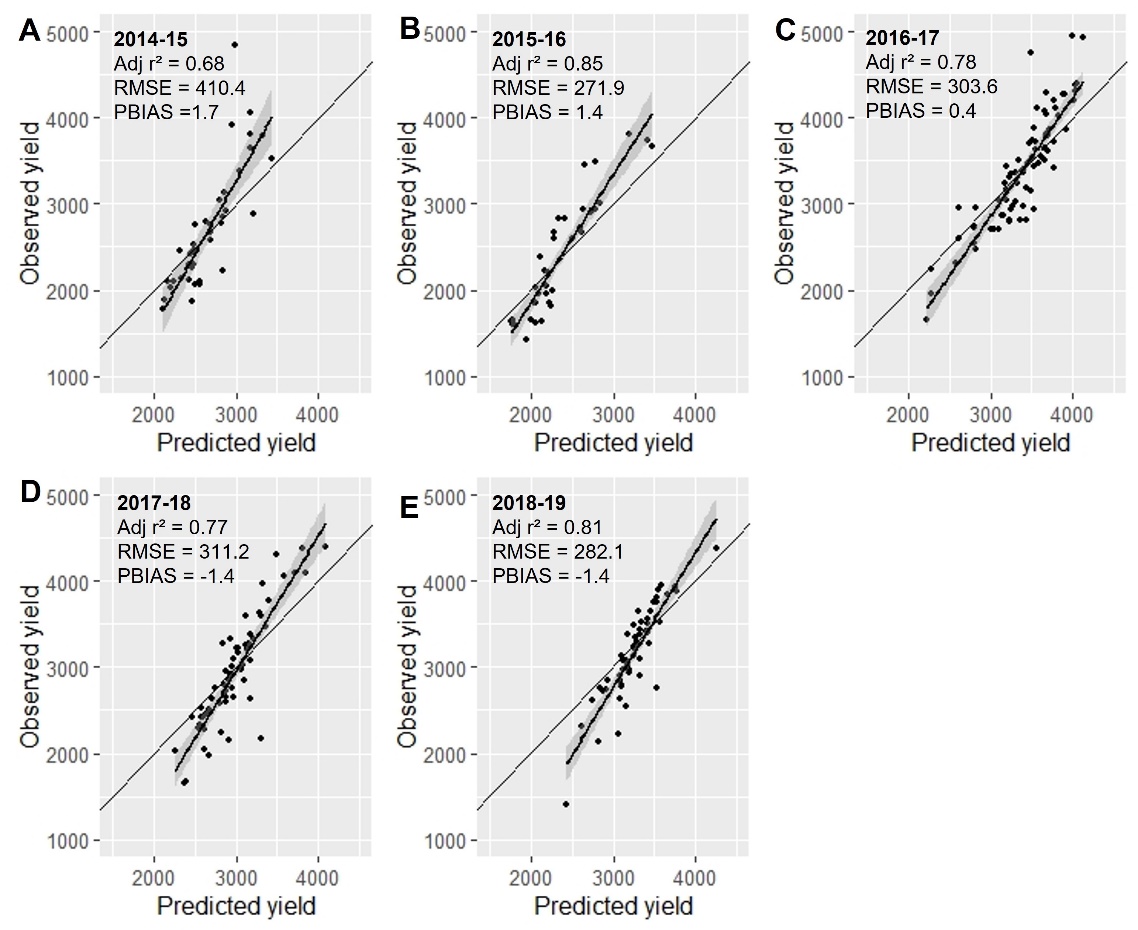
**

**Figure S1.** Random forest estimated yields for each year using Equation 2.


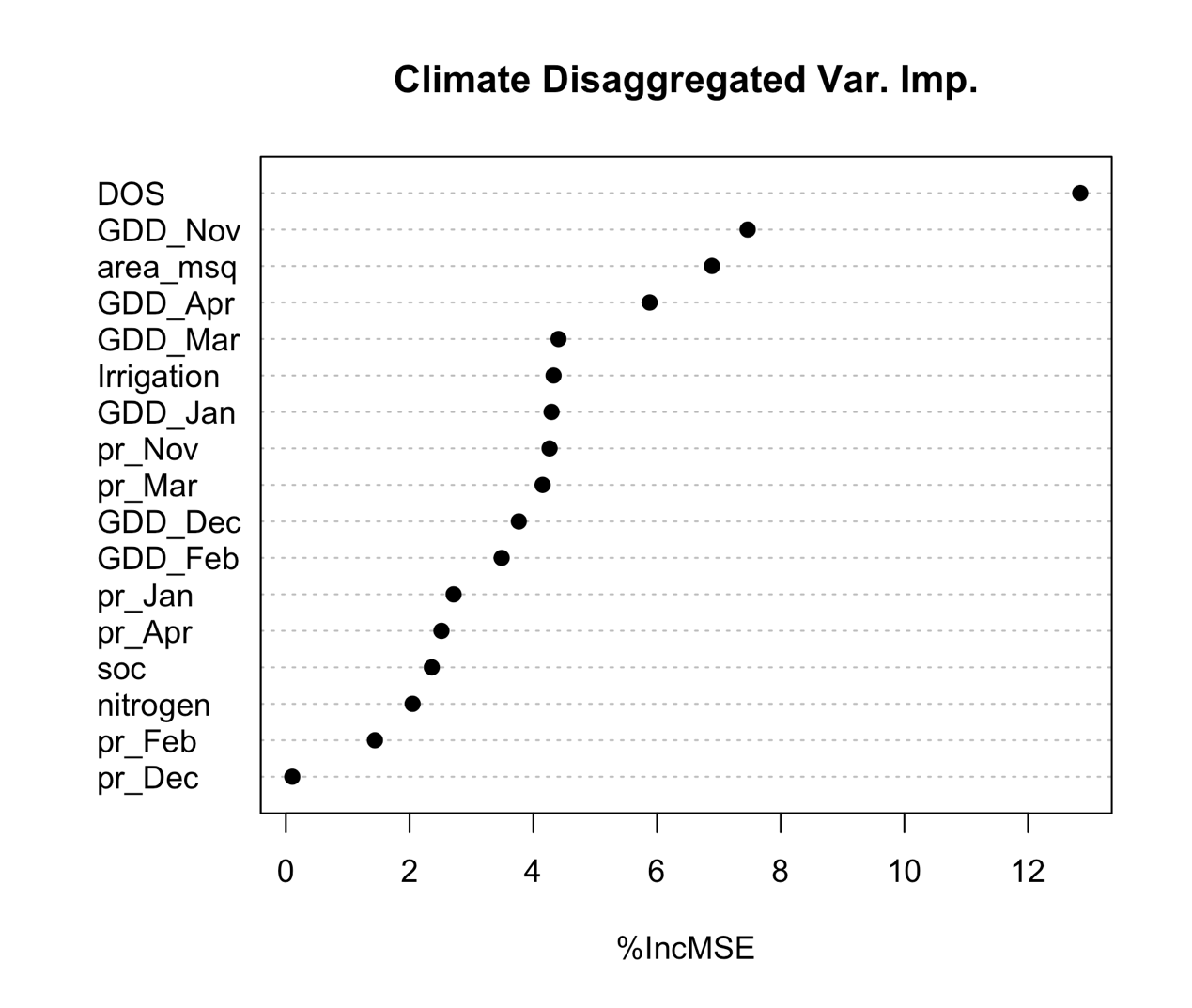


**Figure S2.** Variable importance plots for the management, weather, and biophysical factors considered to explain yield gaps for the years 2014-15 and 2015-16 when using monthly, disaggregated GDD and precipitation data. We find that the importance of sow date (DOS) and irrigation (Irrigation) are similar with the model that uses seasonal data (Figure 5).


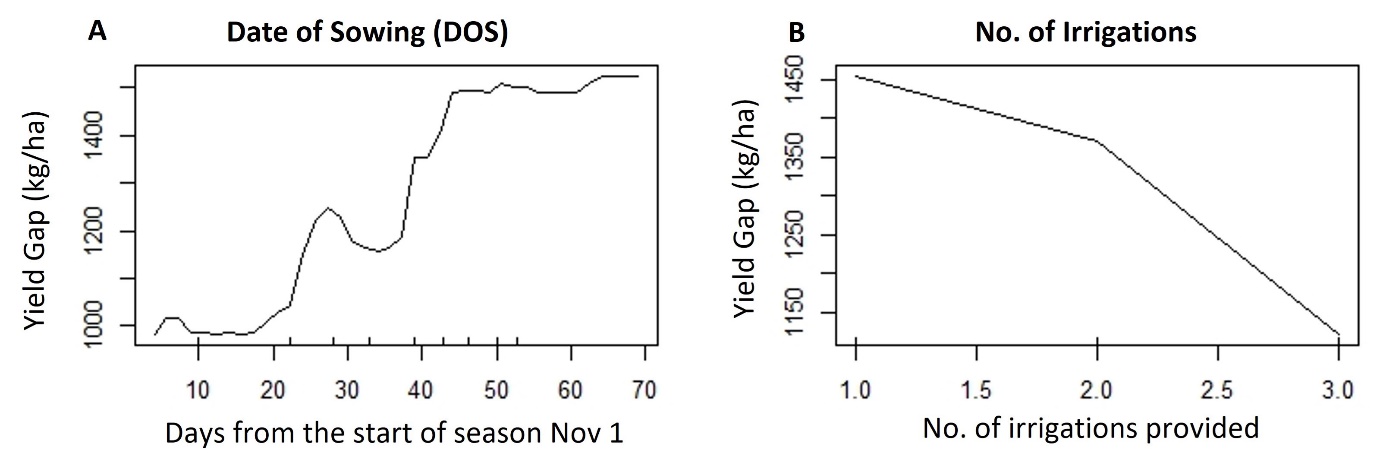


**Figure S3.** Partial dependence plots showing the relationship between yield gap and date of sowing (Panel A) and the number of irrigations (Panel B) for 2014-15 and 2015-16.

**Table S1.** Number of fields where crop cuts were collected each year.

| **Year** | **Number of Fields** | **Size of the sub plot** | **No. of subplots per field** |
| --- | --- | --- | --- |
| 2014-15 | 38 | 2 × 1m² | 3 |
| 2015-16 | 36 | 2 × 1m² | 3 |
| 2016-17 | 79 | 2 × 1m² | 2 |
| 2017-18 | 60 | 2 × 1m² | 2 |
| 2018-19 | 58 | 5 × 1m² | 1 |

**Table S2. Type of sensor and dates of images used per year for satellite yield estimation.**

| **Year** | **Sensor** | **Image Dates** |
| --- | --- | --- |
| 2014-15 | SkySat | Feb 18, Mar 11, Mar 22, Mar 23, Mar 28, Apr 6 |
| 2015-16 | SkySat | Dec 21, Jan 3, Feb 11, Mar 16, Apr 4 |
| 2016-17 | PlanetScope | Jan 13, Feb 25, Mar 12, Apr 14 |
| 2017-18 | PlanetScope | Nov 21, Feb 11, Feb 21, Feb 27, Mar 9, Mar 17, Mar 24, Mar 30, Apr 4, Apr 16 |
| 2018-19 | PlanetScope | Nov 8, Nov 17, Jan 29, Feb 10, Mar 4, Mar 11, Mar 27, Apr 3, Apr 12 |

**Table S3.** Variance Inflation Factors (VIF) for each variable considered in our random forest regression models. VIF values greater than 5 suggest that a given variable is correlated with others in the model.

| **2014-15** | | **2015-16** | | | **2016-17** | | | **2017-18** | | | **2018-19** | |
| --- | --- | --- | --- | --- | --- | --- | --- | --- | --- | --- | --- | --- |
| **Var.** | **VIF** | **Var.** | **VIF** | **Var.** | | **VIF** | **Var.** | | **VIF** | **Var.** | | **VIF** |
| Feb 18 | 1.3 | Dec 21 | 1.87 | Jan 13 | | 1.68 | Nov 21 | | 1.90 | Nov 8 | | 7.50 |
| Mar 11 | 5.6 | Jan 3 | 3.33 | Feb 25 | | 5.47 | Feb 11 | | 5.23 | Nov 17 | | 3.12 |
| Mar 22 | 43.8 | Feb 11 | 3.51 | Mar 12 | | 5.47 | Feb 21 | | 12.61 | Jan 29 | | 10.28 |
| Mar 23 | 45.3 | Mar16 | 2.18 | Apr 14 | | 1.27 | Feb 27 | | 27.37 | Feb 10 | | 7.84 |
| Mar 28 | 6.6 | Apr 4 | 2.24 |  | |  | Mar 9 | | 31.14 | Mar 4 | | 5.51 |
| Apr 6 | 2.5 |  |  |  | |  | Mar 17 | | 40.29 | Mar 11 | | 7.90 |
|  |  |  |  |  | |  | Mar 24 | | 17.84 | Mar 27 | | 10.63 |
|  |  |  |  |  | |  | Mar 30 | | 5.59 | Apr 3 | | 4.42 |
|  |  |  |  |  | |  | Apr 4 | | 3.58 | Apr 12 | | 2.03 |
|  |  |  |  |  | |  | Apr 16 | | 1.83 |  | |  |

**Table S4.** Results from a 5-fold cross validation analysis, where we used a 70:30 split for training and testing data. We report mean R^2^ values when comparing predicted versus observed yield for training and testing datasets across the 5 folds for each model. R^2^ values are similar between training and testing datasets, suggesting that our random forest models are not overfitting the data. Here, validation was done using yield predictions when using mean GCVI values for each field, and not by aggregating pixel-level yield predictions as was done in Figures 2 and S1.

|  | **2014-15** | **2015-16** | **2016-17** | **2017-18** | **2018-19** |
| --- | --- | --- | --- | --- | --- |
| **Training** | 0.82 | 0.95 | 0.88 | 0.93 | 0.92 |
| **Testing** | 0.19 | 0.91 | 0.87 | 0.96 | 0.94 |

**Table S5.** Descriptive statistics of the yield per year at the field level

|  | **2014** | **2015** | **2016** | **2017** | **2018** |
| --- | --- | --- | --- | --- | --- |
| **Average Yield** | 2826 | 2412 | 3498 | 2853 | 3146 |
| **Median Yield** | 2691 | 2300 | 3577 | 2762 | 3109 |
| **Max Yield** | 6101 | 4171 | 4424 | 4362 | 5383 |
| **Min Yield** | 1792 | 1429 | 1788 | 1985 | 1355 |
| **95%Yp** | 3856 | 3464 | 4093 | 3738 | 3963 |

**Table S6.** Variables used in the study to understand the drivers of yield gaps along with their source, spatial resolution, and previous literature that highlight the importance of the variable for explaining yield variation.

| **Variable** | **Source** | **Resolution** | **Previous Literature** |
| --- | --- | --- | --- |
| DOS – date of sowing | Survey | Field level | Ortiz-Monasterio et al.^1^ |
| Irrigation – number of irrigations applied | Survey | Field level | Zaveri and Lobell^2^ |
| Plotarea – area of the field sampled | Survey | Field level | Taylor et al.^3^ |
| Avg_Temp | Terra Climate  (Abatzoglou et al. 2018)^4^ | Monthly dataset at 1/24°, ~4-km | Aslam et al. ^5^ |
| Tot_Rain – total rainfall (in mm) throughout the growing season | Terra Climate  (Abatzoglou et al. 2018)^4^ | Monthly dataset at 1/24°, ~4-km | Daloz et al.^6^ |
| Nitrogen – amount of soil nitrogen | World Soil Information Service (WoSIS) (Batjes at al. 2020)^7^ | 250m | Ahrens et al.^8^ |
| Soil_Org_C – amount of soil organic carbo | World Soil Information Service (WoSIS) (Batjes at al. 2020)^7^ | 250m | Bharali et al.^9^ |

**Table S7.** Results from an ANOVA analysis that examine whether the type of crop cut protocol used (Table S1) influences satellite yield estimation accuracy. We calculated the dependent variable as the absolute value of the residual between observed crop cut and satellite estimated yield values. We see that the p value is > 0.05, suggesting that crop cut collection protocol does not impact satellite yield estimation accuracy.

|  | Df | Sum Sq | Mean Sq | F Value | P Value |
| --- | --- | --- | --- | --- | --- |
| plots | 2 | 74932 | 37466 | 2.137 | 0.12 |
| Residuals | 267 | 4681006 | 17532 |  |  |

**References**

1. Ortiz-Monasterio, R. J. I., Dhillon, S. S. & Fischer, R. A. Date of sowing effects on grain yield and yield components of irrigated spring wheat cultivars and relationships with radiation and temperature in Ludhiana, India. *Field Crops Res* **37,** 169–184 (1994).

2. Zaveri, E. & B. Lobell, D. The role of irrigation in changing wheat yields and heat sensitivity in India. *Nat. Commun.* **10,** 4144 (2019).

3. Taylor, S. L., Payton, M. E. & Raun, W. R. Relationship between mean yield, coefficient of variation, mean square error, and plot size in wheat field experiments. *Commun. Soil Sci. Plant Anal.* **30,** 1439–1447 (1999).

4. Abatzoglou, J. T., Dobrowski, S. Z., Parks, S. A. & Hegewisch, K. C. TerraClimate, a high-resolution global dataset of monthly climate and climatic water balance from 1958–2015. *Sci. Data* **5,** 170191 (2018).

5. Aslam, M. A., Ahmed, M., Stöckle, C. O., Higgins, S. S., Hassan, F. ul & Hayat, R. Can Growing Degree Days and Photoperiod Predict Spring Wheat Phenology? *Front. Environ. Sci.* **5,** (2017).

6. Daloz, A. S., Rydsaa, J. H., Hodnebrog, Ø., Sillmann, J., van Oort, B., Mohr, C. W., Agrawal, M., Emberson, L., Stordal, F. & Zhang, T. Direct and indirect impacts of climate change on wheat yield in the Indo-Gangetic plain in India. *J. Agric. Food Res.* **4,** 100132 (2021).

7. Batjes, N. H., Ribeiro, E. & van Oostrum, A. Standardised soil profile data to support global mapping and modelling (WoSIS snapshot 2019). *Earth Syst. Sci. Data* **12,** 299–320 (2020).

8. Ahrens, T. D., Lobell, D. B., Ortiz-Monasterio, J., Li, Y. & Matson, P. A. Narrowing the agronomic yield gap with improved nitrogen use efficiency: a modeling approach. *Ecol Appl* **20,** 91–100 (2010).

9. Bharali, A., Baruah, K. K., Bhattacharyya, P. & Gorh, D. Integrated nutrient management in wheat grown in a northeast India soil: Impacts on soil organic carbon fractions in relation to grain yield. *Soil Tillage Res.* **168,** 81–91 (2017).
